# A wheat spike detection method based on Transformer

**DOI:** 10.3389/fpls.2022.1023924

**Published:** 2022-10-20

**Authors:** Qiong Zhou, Ziliang Huang, Shijian Zheng, Lin Jiao, Liusan Wang, Rujing Wang

**Affiliations:** ^1^ Institute of Intelligent Machines, Hefei Institutes of Physical Science, Chinese Academy of Sciences, Hefei, China; ^2^ Science Island Branch, University of Science and Technology of China, Hefei, China; ^3^ College of Information and Computer, Anhui Agricultural University, Hefei, China; ^4^ Department of Information Engineering Southwest, University of Science and Technology, Mianyang, China; ^5^ School of Internet, Anhui University, Hefei, China

**Keywords:** deep learning, IoU loss function, transformer, wheat spike detection, agriculture

## Abstract

Wheat spike detection has important research significance for production estimation and crop field management. With the development of deep learning-based algorithms, researchers tend to solve the detection task by convolutional neural networks (CNNs). However, traditional CNNs equip with the inductive bias of locality and scale-invariance, which makes it hard to extract global and long-range dependency. In this paper, we propose a Transformer-based network named Multi-Window Swin Transformer (MW-Swin Transformer). Technically, MW-Swin Transformer introduces the ability of feature pyramid network to extract multi-scale features and inherits the characteristic of Swin Transformer that performs self-attention mechanism by window strategy. Moreover, bounding box regression is a crucial step in detection. We propose a Wheat Intersection over Union loss by incorporating the Euclidean distance, area overlapping, and aspect ratio, thereby leading to better detection accuracy. We merge the proposed network and regression loss into a popular detection architecture, fully convolutional one-stage object detection, and name the unified model WheatFormer. Finally, we construct a wheat spike detection dataset (WSD-2022) to evaluate the performance of the proposed methods. The experimental results show that the proposed network outperforms those state-of-the-art algorithms with 0.459 mAP (mean average precision) and 0.918 AP_50_. It has been proved that our Transformer-based method is effective to handle wheat spike detection under complex field conditions.

## 1 Introduction

Wheat is one of the most important food crops in the world, with an annual production of 730 million tons in around 215 million ha (Catherine et al., 2014). As the global yield supports approximately 30% of the world population, wheat production estimation has become a focus of agricultural research. It could provide key indicators for agricultural decision-making and field management. Since wheat spike is a major factor that reflects the grain number per unit area, it is significant to accurately detect the wheat spike for estimating crop yield.

Traditional field yield estimation methods are time-consuming, inefficient, and poorly representative, so they are not suitable for current large-scale yield forecasting tasks. With the development of computer vision, many researchers have conducted research through machine learning techniques. Fang et al. (2020) proposed to estimate the wheat tiller density based on terrestrial laser scanning data. Fernandez-Gallego et al. (2019) used zenithal/nadir thermal images to count the number of wheat spikes. Jin et al. (2017) adopted unmanned aerial vehicles (UAVs) to obtain high-resolution imagery for estimating wheat plant density. In these traditional machine learning studies, image texture, geometry, and color intensity are primarily used to discriminate spikes. However, the process is partly manually designed to define the range and threshold in the model. They are not robust enough for different situations with dense distribution, complex structural environments, and severe occlusion in the field (Zhang et al., 2020a). Convolutional neural networks (CNNs) have been introduced into the research of wheat spike detection in recent studies. Khoroshevsky et al. (2021) suggested that a network incorporates multiple targets in a single deep model, and the results show that the method is effective as a yield estimator. Misra et al. (2020) combined digital image analysis with CNN techniques to identify and count wheat spikes. CNNs are effective to extract local information, but they lack the ability to extract long-range features from global information. Due to the field environment of wheat being complex, *i*.*e*., dense distribution, complex structural environment, and severe occlusion, it is hard for CNNs to perform well.

The evolution of Transformer (Vaswani et al., 2017) in natural language processing (NLP) provides an alternative path, and many researchers have subsequently transferred the NLP models to computer vision models. Compared with conventional CNN backbones, Transformers always produce global receptive fields rather than local receptive fields, which is more suitable for detecting objects in complex backgrounds. The Transformer architecture avoids repetition and instead relies entirely on the attention mechanism to map the global dependencies between inputs and outputs. The significant success in the natural language processing domain motivates researchers to investigate the application in classification (Dosovitskiy et al., 2021) and dense prediction tasks (Bochkovskiy et al., 2020; Carion et al., 2020; Xizhou et al., 2020). There are two main challenges in transferring the NLP Transformer to the visual domain Transformer. Firstly, unlike the word tokens that are the basic elements of a linguistic Transformer, the vision elements can be very different from the NLP in scale. Another is that Transformer has high computational and memory costs for prediction tasks.

Bounding box regression is a key operation to locate the target object in detection tasks. The loss function is to calculate the difference between the regression result and the true value and finally minimize the regression error. The *ln*−*norm* loss function is widely adopted in bounding box regression, while the common *ln*−*norm* loss (e.g. *l*1−*norm* or *l*2−*norm* ) is used for measuring the distance between bounding boxes. However, according to the research of Yu et al. (Yu et al., 2016; Rezatofighi et al, 2019), it is not tailored to the Intersection over Union (IoU) metric. IoU loss (Yu et al., 2016) and generalized IoU (GIoU) loss (Rezatofighi et al., 2019) have recently been suggested to improve the IoU metric. IoU loss can be effective only when the bounding boxes overlap, but it is useless for non-overlapping cases. GIoU adds a penalty term that the predicted bounding box will move to the target box without overlapping. Nevertheless, GIoU empirically has a lower convergence speed, and it will degrade to IoU loss for enclosing boxes (Zheng et al., 2020). Therefore, it is important to design an effective loss function for bounding box regression.

**Figure 1:**
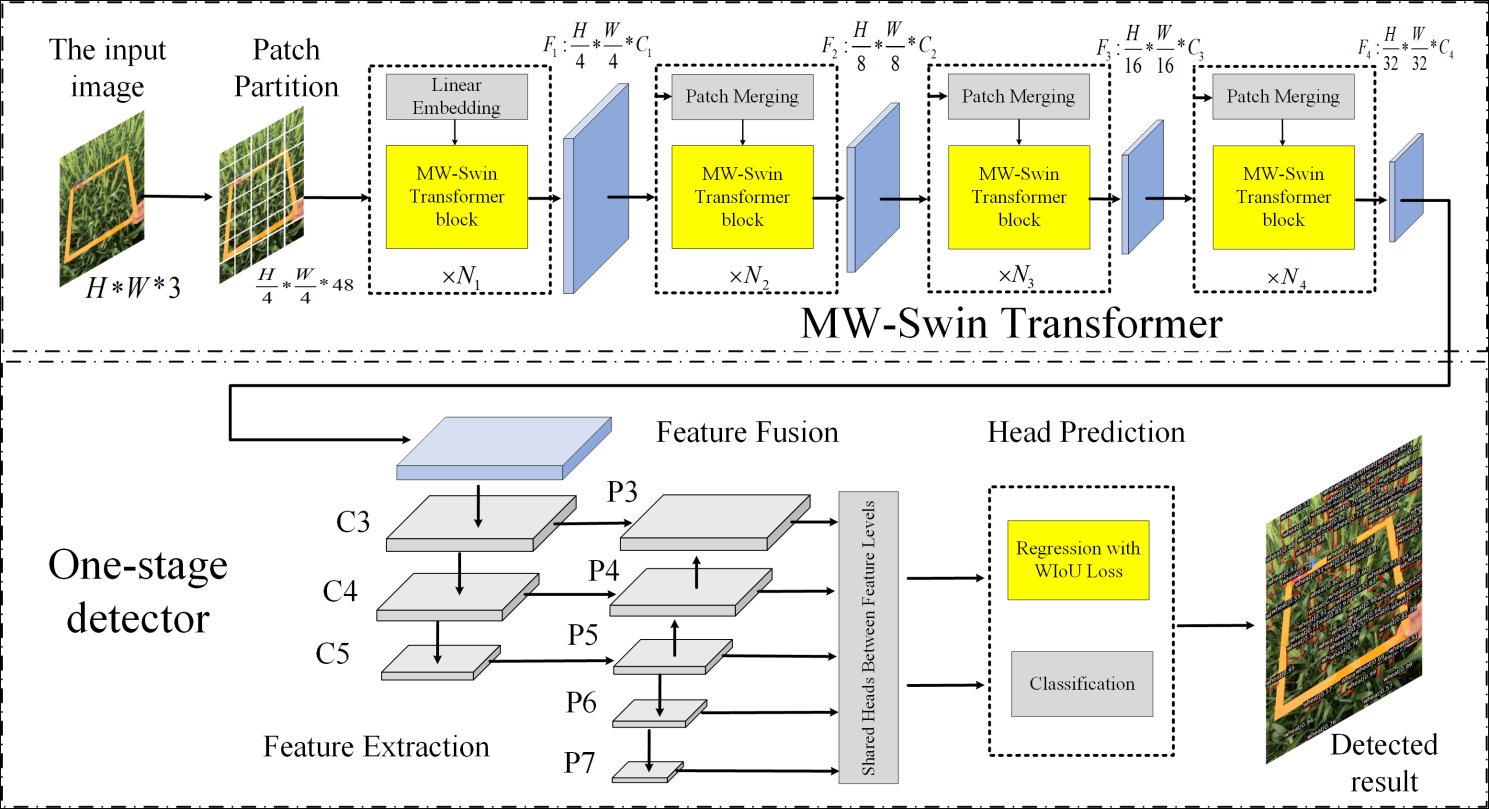
The main architecture of WheatFormer.

In this work, we aim to explore a Transformer-based network for wheat spike detection. To the best of our knowledge, this is the first attempt using Transformer in the wheat detection field. Inspired by the novel architecture of Swin Transformer (Liu et al., 2021) and exploring to overcome the above-mentioned limitations, we propose a Transformer-based network named MW-Swin Transformer. It has the following advantages: Firstly, compared with the conventional Transformer, the proposed Transformer occupies the hierarchical architecture that is essential for downstream tasks. Secondly, compared with Swin Transformer, we inherit the excellent network and design of a multi-window Transformer block to extract target features with different scales. Thirdly, our method has three variants according to the number of stacked layers, which is flexible to fit the actual requirements. Furthermore, we propose a WIoU loss for bounding box regression. Specifically, we add a penalty term on IoU loss, considering the overlap area, Euclidean distance, and aspect ratio. The three geometric indicators are important, *e*.*g*., the Euclidean distance is used to minimize the distance of central points in two bounding boxes, and the consistency of aspect ratios is also bringing about an impact on IoU loss. We incorporate the proposed methods into the FCOS and name the new model WheatFormer, as illustrated in **Figure 1**. WheatFormer contains two major parts: the multi-window Swin (MW-Swin) Transformer and the wheat detector. The input image is split into non-overlapping patches, and each patch is regarded as a token and fed into the MW-Swin Transformer backbone to learn long-range features from global information. Then, the extracted feature maps are fed into the one-stage detector to locate the wheat spike. Finally, we construct a wheat spike detection dataset named WSD-2022 to evaluate the performance of the proposed WheatFormer. The dataset contains 6,404 images from two data sources, the first was from the Global Wheat Head Detection (GWHD) dataset (David et al., 2021) and the second was collected in the field environment by our collaborators. The major contributions of our work are as follows:

● We propose the MW-Swin Transformer with multiple windows for different scale objects, which inherits from the shifted windows in Swin Transformer. This strategy brings a much lower latency than those previous Transformer models, leading to strong performance due to the global receptive field.
● A WIoU loss function is proposed for bounding box regression, considering three important geometric indicators. WIoU helps the network achieve a better performance than normal IoU loss and other improved IoU loss functions.
● We build the WSD-2022 dataset for detecting wheat spikes. This dataset contains wheat spike images from different regions and different developmental stages. Our work provides a richer benchmark dataset for wheat spike detection tasks.

## 2 Related work

### 2.1 CNN-based methods in wheat spike detection

CNNs have been widely used in computer vision tasks, such as image classification (Huang et al., 2017), object detection (Ren et al., 2017), and semantic segmentation (He et al., 2017), which have achieved excellent achievements. Differently from traditional machine learning methods, CNNs can automatically abstract features without manual intervention. Sadeghi-Tehran et al. (2019) proposed a low-computational-cost system to automatically detect the number of wheat spikes, which used simple linear iterative clustering with CNN. Hasan et al. (2018) introduced a robust R-CNN model for the accurate detection, counting, and analysis of wheat ears for yield estimation. Wang et al. (2019) provided a method based on a fully convolutional network and Harris corner detection, solving the problem of counting wheat ears in field conditions. Madec et al. (2019) used Faster R-CNN to provide accurate ear density using RGB images taken from the UAV. Pound et al. (2017) investigated a deep learning method capable of accurately localizing wheat ears and spikelets. Gong et al. (2020) proposed a novel object method of wheat head detection based on dual SPP networks to enhance the speed and accuracy of detection. Yang et al. (2021) combined the convolutional neural network and attention mechanism technology to propose a CBAM-YOLOv4 wheat ear detection and counting method.

### 2.2 Object detection

Object detection methods can be divided into two groups: with two stages and with one-stage. For two-stage detectors, the first stage is to produce lots of high-quality region proposals by a proposal generator, and the second stage is classifying and refining the proposals by region-wise subnetworks. R-CNN (Girshick et al., 2014) and Fast R-CNN (Girshick, 2015) are the typical networks of two-stage detectors, which combined the region proposals and CNN for object detection. Faster R-CNN (Ren et al., 2017) was proposed to speed up Fast R-CNN and promote detection accuracy by using region proposal network. Other two-stage detectors mainly include Mask R-CNN (He et al., 2020), Libra R-CNN (Pang et al., 2019), and Cascade R-CNN (Cai and Vasconcelos, 2018). However, two-stage detectors show a weakness in detection efficiency (Redmon et al., 2016). For one-stage detectors, they drop the process of generation region proposals, treating the object detection task as a single shot problem, such as the YOLO series networks: YOLO (Redmon et al., 2016), YOLOv3 (Redmon and Farhadi, 2018), and YOLOv4 (Bochkovskiy et al., 2020). Tian et al. (2019) proposed a fully convolutional one-stage object detector. This method avoided the complex computation by eliminating the predefined set of region proposals. SSD (Fu et al., 2017) introduced additional context into the popular general object detection.

### 2.3 Vision Transformer

The Transformer is proposed by Vaswani et al. (2017), which is widely used in NLP tasks. Recently, the pioneering work of vision Transformer ViT (Dosovitskiy et al., 2021) demonstrated that the pure Transformer-based model can also achieve competitive performance in vision tasks. Based on the success of ViT, many studies have on designing more advanced Transformer base networks been published, including image processing (Wan et al., 2021), classification (Wang et al., 2021), object detection (Carion et al., 2020), and semantic segmentation (Zheng et al., 2021). However, the normal ViT-based models are not compatible with many downstream tasks due to the high computational cost. To alleviate the limitations, an efficient and effective hierarchical Transformer named Swin Transformer (Liu et al., 2021) was proposed as a unified vision backbone. Swin Transformer designed the shifted windows mechanism, achieving state-of-the-art performance in many downstream tasks. We introduce Swin Transformer due to its excellent characteristics, and the hierarchical architecture is designed to reduce the complex computation by progressively decreasing the shape of feature maps.

## 3 Materials and methods

### 3.1 Dataset

**Figure 2:**
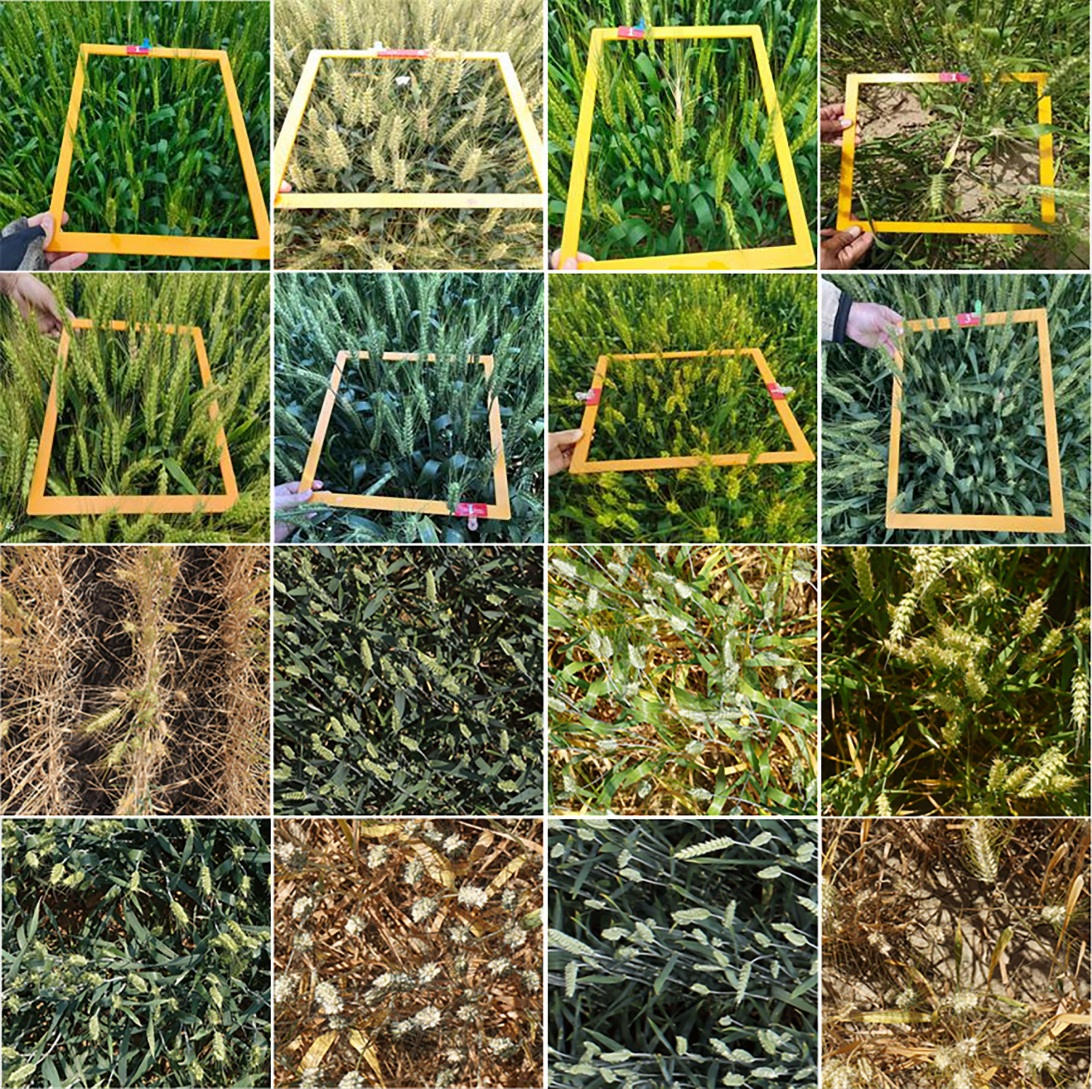
Samples of the WSD-2022 dataset. The first and second rows of the figure show the images that we acquired, while the third and fourth rows of the figure come from GWHD.

We built a wheat spike detection dataset named WSD-2022, containing a total of 6,404 images, of which 978 images we collected ourselves in the field environment. We conducted wheat image collection in four locations, including Dangtu County, Ma’anshan; Feidong County, Hefei; Guizhi District, Chizhou; and Susong County, Anqing. The images were collected from April 18 to May 10, 2021 from the flowering stage to the milk stage of maturity. We collected the wheat spikes of varieties with different colors, shapes, and densities, thus increasing the diversity of the data. We shot the images using different types of cameras at different shooting angles and distances to collect image data under different lighting conditions to enhance the robustness of the model. About 80% of the images were captured at a resolution of over 3,000*4000 pixels. The captured images need to label each wheat spike, and we use LabelImg software to annotate the bounding boxes around the wheat spikes. Each wheat spike is labeled with a bounding box, the annotation is represented as a vector (*x*,*y*,*w*,*h*) where (*x*,*y*) are the coordinates of the upper left and (*w*,*h*) are the width/height of the bounding box. **Figure 2** shows some examples of WSD-2022. Due to the different shooting angles, different lighting conditions, different wheat growth periods, different wheat distribution densities, and different wheat spike sizes, we can find the diversity and complexity of the dataset. We randomly split the WSD-2022 into training and validation subsets at a ratio of 8:2. The details of the two subsets are summarized in **Table 1**.

**Table 1:** Number of images in the WSD-2022 dataset.

WSD-2022	Train	Validation	Total
Ours	782	196	978
GWHD	4,309	1,117	5,426
Total	5,091	1,313	6,404

### 3.2 MW-Swin Transformer

#### 3.2.1 Overall architecture

This section describes the design of MW-Swin Transformer. The pyramid structure was introduced based on the Transformer model to generate hierarchical feature maps for downstream tasks. The overall architecture of MW-Swin Transformer is similar to CNN networks. As shown in (**Figure 1**). For an input image with size of H*W*3 , we follow Swin Transformer to split the image into patches at first (we treat each patch as a “token”); the patch size is 4*4. By such approach, the feature dimension of each patch becomes 4*4*3 = 48. Then, a linear embedding layer is employed to project the feature dimension to arbitrary dimension (set as *C* ). To produce hierarchical feature representation, the model architecture consists of four stages; a patch merging layer is added after each stage for down-sampling (reduce the number of tokens, which is similar to the pooling layer in CNN).

In the first stage, we divide the input image into *HW*/4^2^ patches, with a size of 4*4*3 for each of them. Through the linear embedding layer, we feed the flattened patches to MW-Swin Transformer blocks (the number of blocks is represented by *N* ), and the output is reshaped to a feature map with a size of *H*/4*W/4*C_1_ (represented as *F*_1_ ). The patch merging layer down-sampled each feature map *F*_*i*_,*i*={1,2,3,4} with strides [4, 8, 16, 32] with respect to the size of the input image. The output dimensions of *F*_*i*_ is set to *C*_*i*_,*i*={1,2,3,4} . Therefore, the output resolution of each stage is *H*/4*W/4*C_1_ , *H*/8*W/8*C_2_ , *H*/16*W/16*C_3_ , and *H*/32*W/32*C_4_ , respectively. With the hierarchical structure, our model possesses the progressive shrinking strategy that adjusts the output scale of each stage so that we can easily apply the model to downstream tasks.

#### 3.2.2 MW-Swin Transformer block

Transformer obtains the powerful ability of long-range context modeling, but the computation complexity of conventional Transformer is quadratic to feature map size. For dense prediction tasks with high-resolution images as input, using conventional Transformer is expensive. Therefore, Swin Transformer is proposed to perform self-attention by non-overlapping local windows and shifted windows. However, the window size of Swin Transformer is fixed, which is not conducive to detecting objects of different sizes. To enlarge the receptive field and obtain global self-attention more flexibly, we propose the MW-Swin Transformer; the architecture is similar to the feature pyramid network, using different-sized windows to detect objects across a large range of scales.

**Figure 3:**
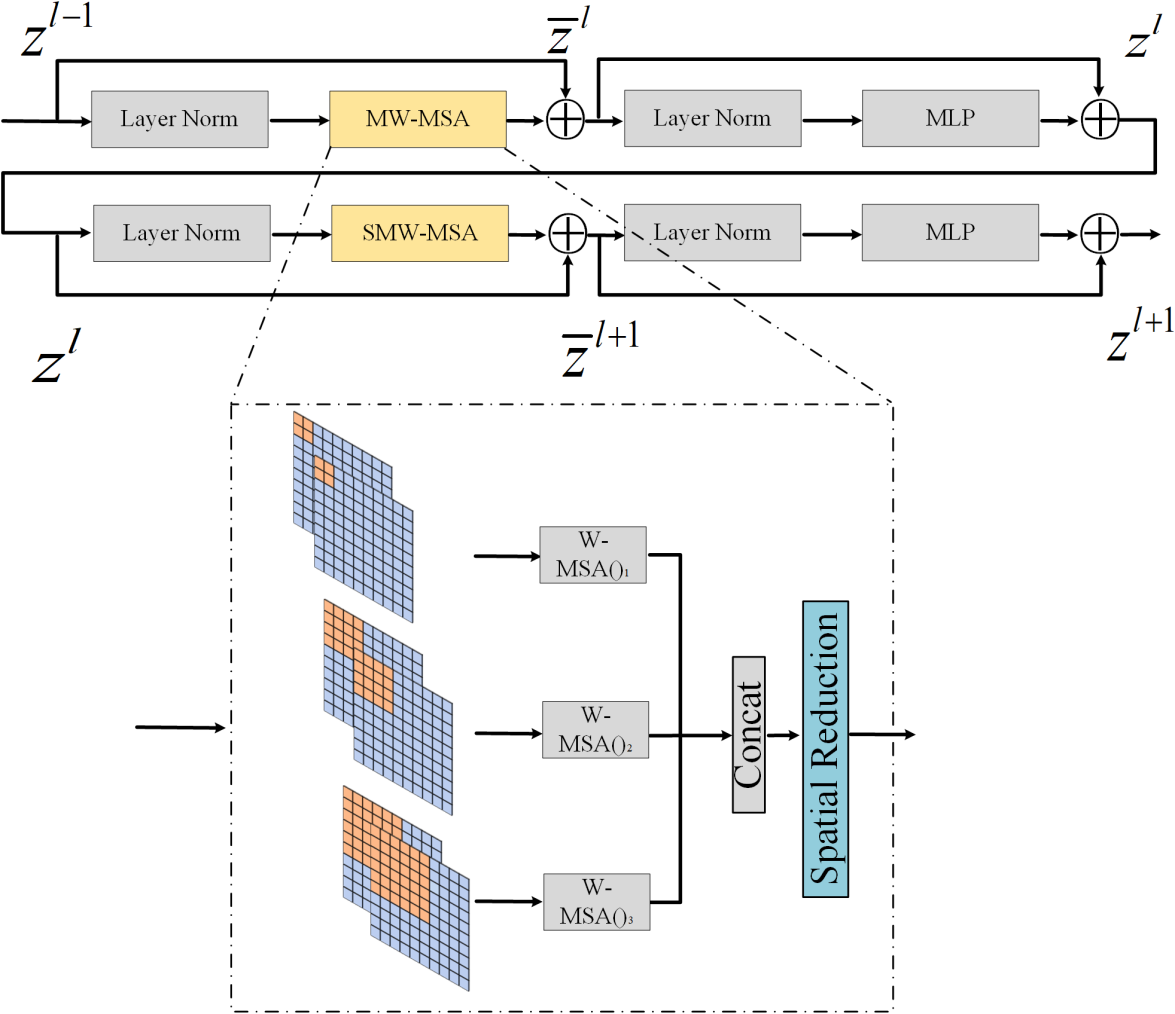
MW-Swin Transformer block.

As shown in **Figure 3**, two consecutive MW-Swin Transformer blocks are presented. Each block contains two LayerNorm (Bosilj et al., 2020) layers, a multi-head self-attention (MSA), and a multilayer perceptron (MLP). The multi-window MSA (MW-MSA) and the shifted multi-window MSA (SMW-MSA) are adopted in the consecutive Transformer blocks, respectively. With the MW-MSA module and the SMW-MSA module, consecutive MW-Swin Transformer blocks can be represented as:


(1) $$\begin{array}{l} {\bar{z}}^{l}=MW-SMA(LN(z^{l-1}))+z^{l-1}\\ {\bar{z}}^{l}=SR({\bar{z}}^{l})\\ z^{l}=MLP(LN({\bar{z}}^{l}))+{\bar{z}}^{l}\\ {\bar{z}}^{l+1}=SMW-SMA(LN(z^{l}))+z^{l}\\ {\bar{z}}^{l+1}=SR({\bar{z}}^{l+1})\\ z^{l+1}=MLP(LN({\bar{z}}^{l+1}))+{\bar{z}}^{l+1} \end{array}$$

where ${\bar{z}}^{l}$ and *z* ^*l*^ represent the outputs of (S)MW-SMA module and the MLP for the block, respectively. MW-MSA equals *Concat*(*W*−*MSA*(*z* ^*l*−1^)_1_,*W*−*MSA*(*z* ^*l*−1^)_2_,*W*−*MSA*(*z* ^*l*−1^)_3_) , where *W*−*MSA*(•)_*i*_,*i*=1,2,3 indicates the *i*_*th*_ window with size *X* , and we set *X*=[7,9,11] in experiments. *SR*(•) denotes the spatial reduction module to reduce the spatial scale of ${\bar{z}}^{l}$ , which reduces the memory and computational cost. Similar to the conventional Transformer (Dosovitskiy et al., 2021; Liu et al., 2021), the attention operation can be computed as follows:


(2) $$Attention(Q,K,V)=Soft\max(\frac{QK^{T}}{\sqrt{d}}+B)V$$

where *Q*,*K*,*V* represent the query, key, and value matrices; the other parameters are in accordance with Swin Transformer.

Compared with the previous MSA in vision Transformers, the MW-MSA controls the computation area in multi-window as a unit. It reduces the complexity and computational cost, enhancing the ability to detect multi-scale features. MW-Swin Transformer block can serve as a plug-and-play block to replace the raw Transformer block in Swin Transformer, with only minor modifications to the vanilla structure.

#### 3.2.3 Architecture variants

We named the base model WheatFormer-B, which is a trade-off between efficiency and accuracy. Considering higher efficiency needs in some cases, we have introduced a small version named WheatFormer-S. On the other hand, when accuracy needs to be considered more, we have introduced a large version named WheatFormer-L. The architectures of our base model and variants are listed in **Table 2**.

**Table 2:** Detailed settings of WheatFormer variants.

Models	*C*_1_,*C*_2_,*C*_3_,*C*_4_	*N*_1_,*N*_2_,*N*_3_,*N*_4_	#Head	#Expansion	#Params (MB)
WheatFormer-S	[96, 192, 384, 768]	[2, 2, 2, 2]	32	*α*=4	42.4
WheatFormer-B	[96, 192, 384, 768]	[2, 2, 6, 2]	32	*α*=4	60.1
WheatFormer-L	[96, 192, 384, 768]	[2, 2, 18, 2]	32	*α*=4	100.6

C_i_ , channel number of the hidden layers in each stage; N_i_ , layer numbers in each stage; #Head, query dimension of each head; #Expansion, expansion layer of each multilayer perceptron; #Params, amount of model parameters.

### 3.3 Wheat detector

#### 3.3.1 One-stage object detector

**Figure 4:**
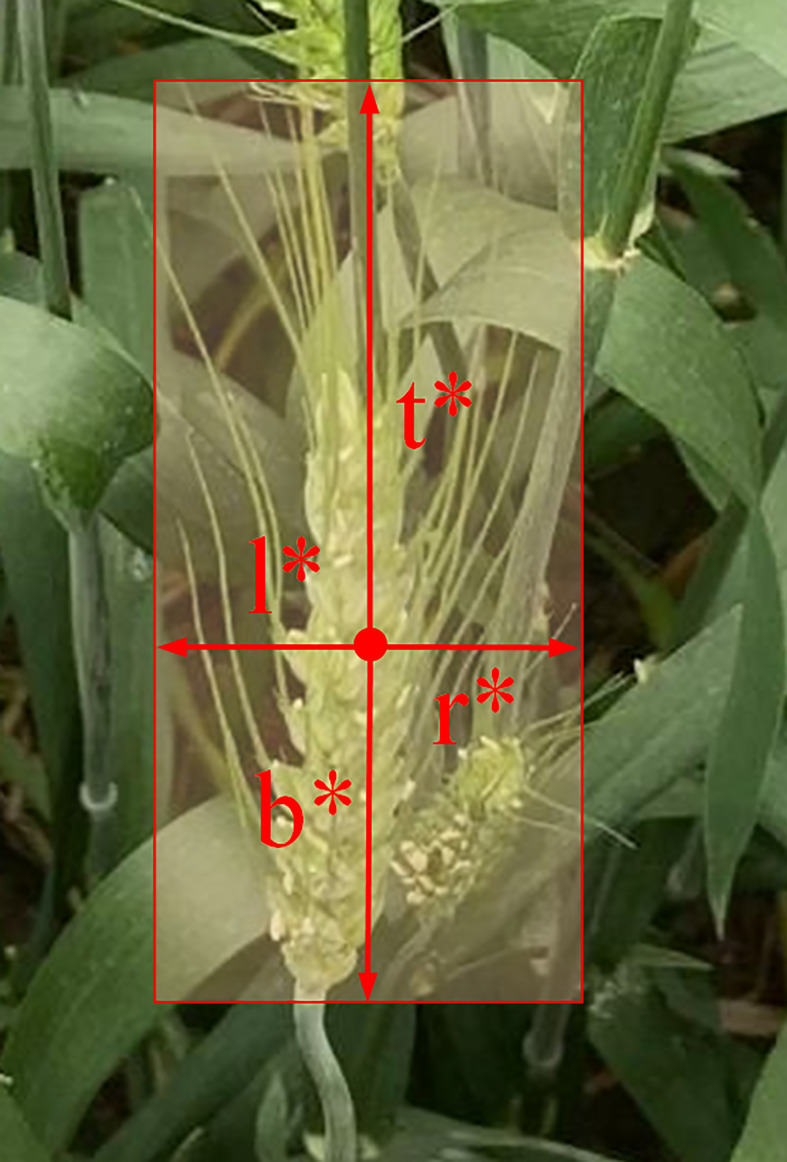
Regression method of FCOS. l*, t*, r*, and b* represent the distances from the pixel to the left, top, right, and bottom, respectively, of the bounding box.


FCOS is a one-stage anchor-free object detection algorithm (Tian et al., 2019) with higher accuracy and faster speed compared with the representative model Faster R-CNN (Ren et al., 2017) and other two-stage detectors. FCOS mainly consists of three parts: a feature extraction backbone, a feature pyramid network (FPN), and a detection head. The backbone extracts multi-level features of the input image. Then, low-level spatial information and high-level semantic information are fed into FPN, generating multi-scale feature maps. In previous research, low-level information can obtain more detailed texture information, which leads to more efficient detection. High-level information gets more semantic information and is more suitable for classification. FCOS is a pixel-based detector, which means that each pixel on the feature map is used for regression. First, each pixel map back to the original input image, and a pixel considers a positive sample if its location falls within any ground-truth box with the correct class label. Otherwise, it is a negative sample. As for regression, FCOS uses a vector *t* ^*^=(*l* ^*^,*t* ^*^,*r* ^*^,*b* ^*^) , where *l* ^*^,*t* ^*^,*r* ^*^,*b* ^*^ denote the distances from the location (*x*,*y*) to the four sides of the bounding box, as shown in **Figure 4**. The target regression process can be formulated as follows:

(3) $$\begin{array}{l} l^{*}=x-x_{0}^{\left(i\right)}\\ t^{*}=y-y_{0}^{\left(i\right)}\\ r^{*}=x_{1}^{\left(i\right)}-x\\ b^{*}=y_{1}^{\left(i\right)}-y \end{array}$$

where (*x*_0_^(*i*)^,*y*_0_^(*i*)^) and represent coordinates of the left-top and right-bottom corners of the bounding box.

#### 3.3.2 WIoU loss

The training loss function of the proposed WheatFormer mainly obtains three branch loss functions:


(4) $$L_{WheatFormer}=\frac{1}{N_{pos}}L_{cls}+\frac{\lambda_{1}}{N_{pos}}L_{\text{center}-ness}+\frac{\lambda_{2}}{N_{pos}}L_{\text{reg}}$$

where *L*_*cls*_ and *L*_center−*ness*_ represent the classification and center-ness loss function which are designed in FCOS. *N*_*pos*_ denotes the number of positive pixels. *λ*_1_ and *λ*_2_ are balance weights to adjust the proportions of three branch loss functions. The parameters follow the settings in Tian et al. (2019). FCOS uses IoU loss to calculate the regression loss, which can be formulated as follows:


(5) $$L_{\text{reg}}=\sum_{x,y\in (R^{p}\cup R^{n})}\left(1-IoU({\mathrm{Pr}}^{x,y},Gt^{x,y})\right)$$

where *R* ^*p*^ represents the positive sample region and *R* ^*n*^ denotes the negative sample region. *Gt* ^*i*,*j*^ indicates the ground truth localization of the pixel (*x*,*y*) , while *Pr* ^*i*,*j*^ denotes the predicted target of (*x*,*y*) .

The IoU loss regresses all bound variables as a whole for joint regression and directly enforces the maximum overlap between the prediction bounding box and the ground truth. The IoU loss leads to faster convergence and more accurate localization compared with the *ln*−*norm* loss used in previous studies. However, the IoU loss cannot provide moving gradients for non-overlapping cases, *i*.*e*., IoU loss is only valid when the bounding boxes overlap. Based on previous researches and the IoU loss, we consider three important geometric metrics, which are the overlap region, Euclidean distance, and aspect ratio of bounding boxes. In summary, we add a penalty term to the IoU loss, named WIoU loss. The new loss function directly minimizes the Euclidean distance between the predicted box and the ground truth. At the same time, we take into account the effect of the consistency of aspect ratios. The WIoU loss function is defined as follows:


(6) $$\begin{array}{l} L_{\text{reg}}=\sum_{x,y\in (R^{p}\cup R^{n})}\left(1-IoU({\mathrm{Pr}}^{x,y},Gt^{x,y})+\psi {║{\mathrm{Pr}}^{x,y},Gt^{x,y}║}_{2}\right)\\ \psi =\frac{4}{\pi^{2}}{\left(\arctan\frac{w_{Gt}^{x,y}}{h_{Gt}^{x,y}}-\arctan\frac{w_{\mathrm{Pr}}^{x,y}}{h_{\mathrm{Pr}}^{x,y}}\right)}^{2} \end{array}$$

where *ψ* measures the consistency of the aspect ratio and plays the role of regularization for the distance between the predicted bounding box and the target bounding box. *w*_*Gt*_ and *h*_*Gt*_ represent the width and height of the ground truth. *w*_*Pr*_ and *h*_*Pr*_ represent the width and height of the predicted bounding box. The optimization of WIoU loss is the same as the IoU loss.

## 4 Experiments and discussion *AP*

### 4.1 Experimental settings

All the experiments were performed using the Pytorch deep learning frame, and the operation system was Ubuntu 18.04 with CUDA10.1. We use a piece of NVIDIA TITAN RTX GPU, Intel Core i9-9900k CPU with 128GB RAM. Furthermore, we train our model with the AdamW (Loshchilov and Hutter, 2017) optimizer for 24 epochs. The initial learning rate is 1*e*−4 , and the weight decay is 0.05. The settings of comparison networks follow the original settings.

### 4.2 Evaluation metrics

In our experiments, we use the evaluation metrics as the metric definition of the COCO dataset. Average precision ( *AP* ) is the area surrounded by the precision-recall curve. The definition of *AP* is defined as Formula 7. *AP*@50 ( *AP*_50_ ) means the value when IoU is equal to 0.5, *AP*@75 ( *AP*_75_ ) is the *AP* value when the IoU equals 0.75, and the mean *AP* ( *mAP* ) is the threshold of the IoU from 0.5 to 0.95 ( *AP*@[0.5:0.05:0.95] ) with a step size of 0.05.


(7) $$\begin{array}{l} precision=\frac{TP}{TP+FP}\\ recall=\frac{TP}{TP+FN}\\ AP=\int_{0}^{1}precision(recall)d(recall) \end{array}$$

where TP (true positive), FP (false positive), and FN (false negative) represent the number of correctly detected wheat spikes, false detected wheat spikes, and missing detected wheat spikes. At the same time, we use *AP*_*s*_ , *AP*_*m*_ , *AP*_*l*_ defined in the COCO dataset in our experiments, which represent the detection accuracy for different target sizes. Considering that the wheat spike in the dataset occupies a larger proportion of the image, we only apply *AP*_*m*_ (for medium targets) and *AP*_*l*_ (for large targets) as the evaluation metric. In the field of object detection, *AP* metric is widely adopted for evaluating the comprehensive detection performance of the model.

### 4.3 Model performance

**Figure 5:**
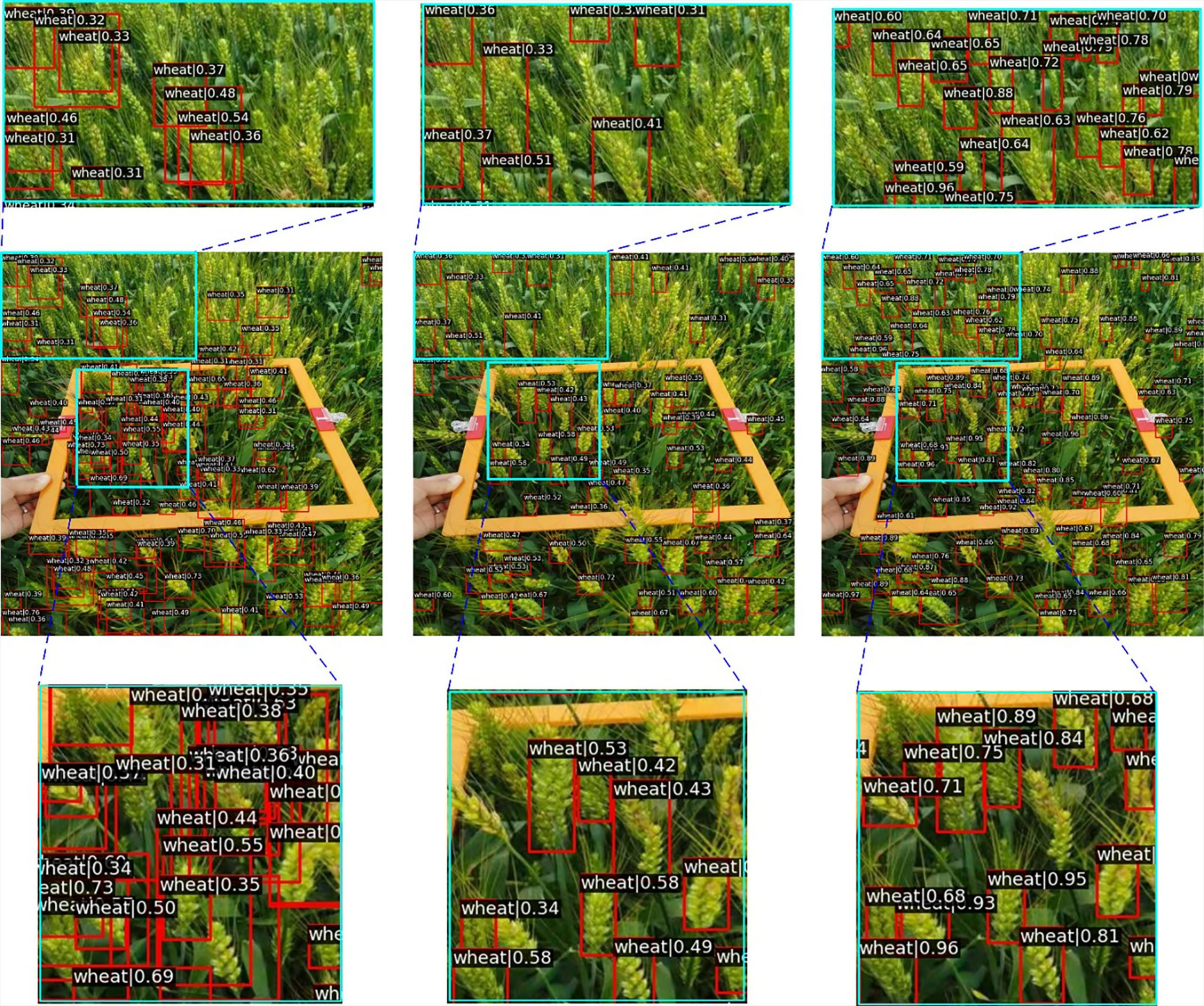
Visualization of the comparative models. The left column represents the result of Faster R-CNN, the middle column represents the result of YOLOv3, and the right column represents the result of WheatFormer.

**Figure 6:**
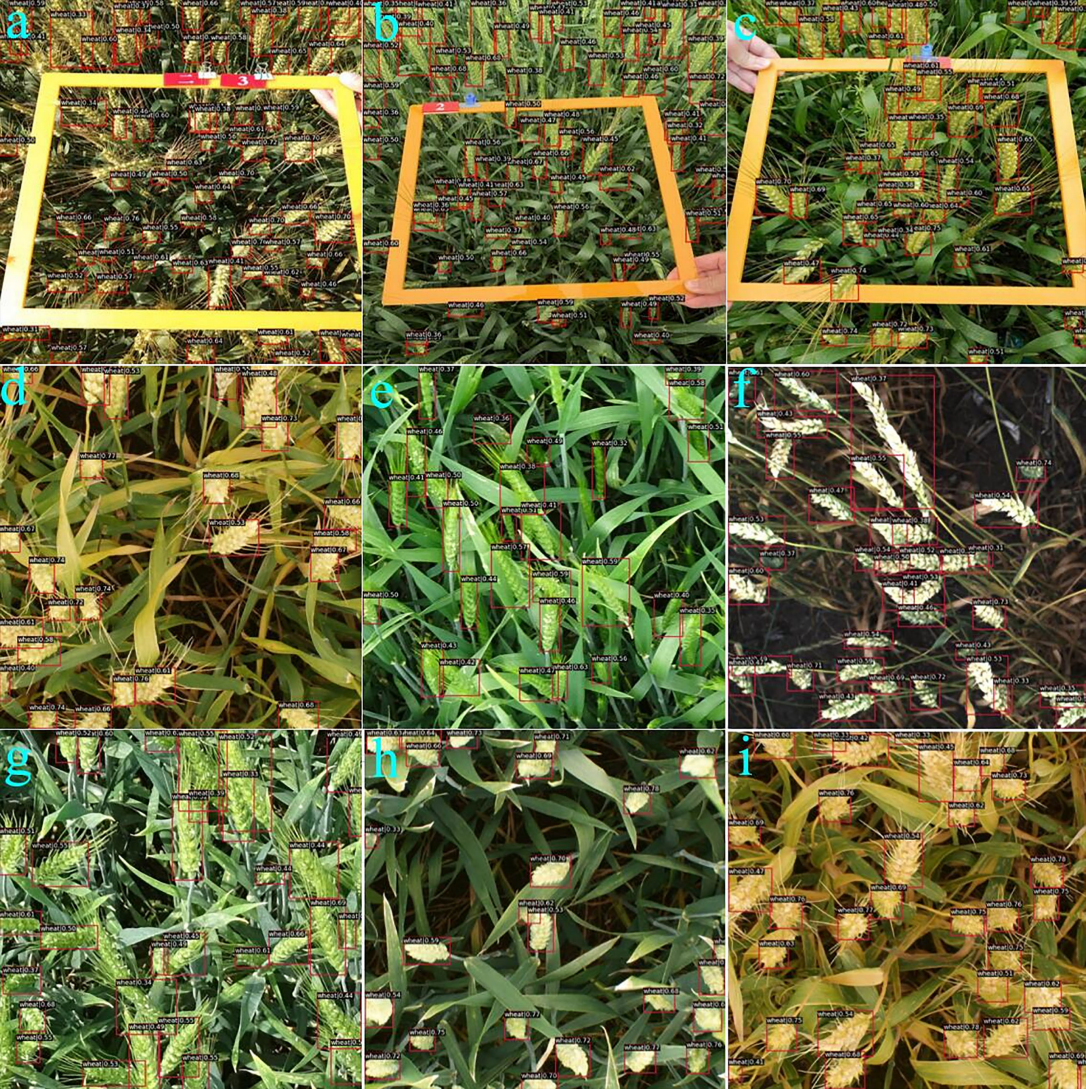
Visualization of detected results by the WheatFormer. **(A)** Early maturity, 65 spikes per image, direct sunlight, and wheat ear group with 80° viewing angle of photographing, **(B)** filling stage, 75 spikes per image, diffuse light conditions, and wheat ear group with 45° viewing angle of photographing, **(C)** filling stage, 45 spikes per image, diffuse light conditions, and wheat ear group with 45° viewing angle of photographing, **(D)** early maturity, 25 spikes per image, diffuse light conditions, and wheat ear group with 90° viewing angle of photographing, **(E)** poplar blossom, 23 spikes per image, direct sunlight, and wheat ear group with 45° viewing angle of photographing, **(F)** the milk stage of maturity, 30 spikes per image, direct sunlight, and wheat ear group with 90° viewing angle of photographing, **(G)** poplar blossom, 27 spikes per image, direct sunlight, and wheat ear group with 30° viewing angle of photographing, **(H)** the milk stage of maturity, 22 spikes per image, diffuse light conditions, and wheat ear group with 90° viewing angle of photographing, and **(I)** the milk stage of maturity, 30 spikes per image, diffuse light conditions, and wheat ear group with 90° viewing angle of photographing.

The experiments in this section aim to demonstrate the effectiveness of the proposed method in terms of detection performance. We compared seven state-of-the-art algorithms, including Faster R-CNN (Madec et al., 2019), Mask R-CNN (He et al., 2020), FCOS (Tian et al., 2019), ATSS (Zhang et al., 2020b), SSD (Fu et al., 2017), Centernet (Zhou et al., 2019), and YOLOv3 (Redmon and Farhadi, 2018). Faster R-CNN and Mask R-CNN are two-stage networks, and the rest are one-stage networks. The experimental results are listed in **Table 3**, and we can find that the proposed WheatFormer outperforms the other models. To be specific, compared with the two-stage CNN-based models, WheatFormer achieves about 10–20% higher in *AP*_50_ and 8–15% improvement in. Compared with the one-stage CNN models, our model increases the *AP*_50_ and *mAP* by 1.2–11.5 and 2.2–9.5%, respectively. In terms of Swin Transformer-based models, the detection performance is generally better than the CNN-based models. The FCOS-based Swin Transformer achieves a *mAP* of 0.452, while our model increases *mAP* by 0.7% and *AP*_50_ by 3.2%. The Mask R-CNN based on Swin Transformer achieves the *AP*_50_ of 0.914, which is comparable to that of WheatFormer, but our model gets a higher *mAP* of 3.3%. Considering the model parameters, our model achieves a larger size than most CNN models but is similar to Swin Transformer-based models. We show some comparison examples in **Figure 5** and the detection results of WheatFormer in **Figure 6**. **Figure 5** shows that Faster R-CNN has too many overlapping prediction boxes, and YOLOv3 obtains too many missing boxes. At the same time, WheatFormer obtains a higher accuracy than the comparison models in classification. In **Figure 6**, we can find that WheatFormer has excellent detection performance at different shooting angles, different light conditions, different wheat growth periods, different wheat distribution densities, and different wheat spikes sizes. WheatFormer can accurately identify most wheat spikes even at high density and high occlusion. This intuitively illustrates the excellent performance of WheatFormer.

**Table 3:** Detection results on WSD-2022.

Method	Backbone	*mAP*	*AP*_50_	*AP*_75_	*AP*_*m*_	*AP*_*l*_	#Params (MB)
Faster R-CNN	ResNet50	0.301	0.709	0.215	0.284	0.339	39.4
Mask R-CNN		0.345	0.774	0.237	0.311	0.382	41.9
Faster R-CNN	ResNet101	0.304	0.750	0.208	0.306	0.352	57.6
Mask R-CNN		0.366	0.812	0.246	0.331	0.394	60.1
FCOS	ResNet50	0.368	0.825	0.250	0.355	0.409	30.6
ATSS		0.364	0.803	0.255	0.357	0.402	30.6
SSD	SSDVGG	0.428	0.890	0.362	0.382	0.488	22.7
CenterNet	ResNet18	0.414	0.876	0.318	0.345	0.487	13.8
YOLOv3	DarkNet53	0.437	0.906	0.381	0.387	0.497	58.7
Faster R-CNN	Swin Transformer	0.397	0.881	0.276	0.352	0.450	65.6
Mask R-CNN		0.426	0.914	0.318	0.379	0.473	68.1
FCOS		0.452	0.886	0.402	0.415	0.523	43.8
WheatFormer	MW-Swin Transformer	**0.459**	**0.918**	**0.384**	**0.415**	**0.533**	**60.1**

Faster R-CNN and Mask R-CNN are the representative models of two stages. FCOS, ATSS, SSD, CenterNet, and YOLOv3 are the representative models of one stage.

### 4.4 Ablation experiments

As mentioned, the major drawbacks of CNN models are the consistently produced local receptive fields, which are unsuitable for detecting objects in complex backgrounds. There are relatively few studies on Transformers-based backbone applied to wheat spike detection. We conduct ablation experiments to represent the effectiveness of our proposed methods.

#### 4.4.1 Effect of the MW-Swin Transformer

**Figure 7:**
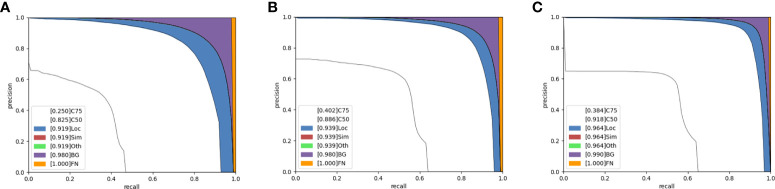
Precision–recall (PR) curves of WheatFormer with different backbones. **(A)** WheatFormer with convolutional neural network backbone. **(B)** WheatFormer with Swin Transformer backbone. **(C)** WheatFormer with MW-Swin Transformer backbone. C75: PR at threshold equals 0.75; C50: PR at threshold equals 0.50; Loc: PR at threshold equals 0.1, and location errors ignored without duplicate detections; Sim: PR after supercategory false positives are removed; Oth: PR after all class confusions are removed; BG: PR after all background false positive are removed; FN: PR after all remaining errors are removed.

In this part, we describe the effectiveness of the proposed MW-Swin Transformer. The results are listed in **Table 4**, which contains three backbones: the CNN backbone, the Swin Transformer backbone, and the MW-Swin Transformer backbone. Obviously, the Swin Transformer backbone-based models greatly improve the detection performance of the state-of-the-art algorithms. For a detailed representative comparison of different backbones, we show the precision–recall curve of WheatFormer in **Figure 7**. Specifically, compared with the CNN backbone and the Swin Transformer backbone, the WheatFormer boosts the Loc, Sim, Oth, and BG to 0.964, 0.964, 0.964, and 0.990. It obtains 9.1% improvements on *mAP* and 9.3% improvements on *AP*_50_ after replacing the backbone with MW-Swin Transformer. This indicates that the proposed Transformer can effectively increase the detection ability of the detectors.

**Table 4:** Comparison of different backbones.

Method	CNN backbone	Swin Transformer	MW-Swin Transformer		*AP*_50_	*AP*_75_
Faster R-CNN	**✔**			0.301	0.709	0.215
		**✔**		0.397 (9.6%↑)	0.881 (17.2%↑)	0.276 (6.1%↑)
			**✔**	0.417 (2%↑)	0.893 (1.2%↑)	0.315 (1.2%↑)
Mask R-CNN	**✔**			0.345 *mAP*	0.774	0.237
		**✔**		0.426 (8.1%↑)	0.914 (14%↑)	0.318 (8.1%↑)
			**✔**	0.433 (0.7%↑)	0.909 (0.5%↓)	0.344 (2.6%↑)
Centernet	**✔**			0.414	0.876	0.318
		**✔**		0.436 (2.2%↑)	0.913 (3.7%↑)	0.372 (5.4%↑)
			**✔**	0.448 (1.2%↑)	0.912 (0.1%↑)	0.365 (0.7%↓)
WheatFormer	**✔**			0. 368	0.825	0. 250
		**✔**		0. 452 (8.4%↑)	0. 886 (6.1%↑)	0. 402 (15.2↑)
			**✔**	**0. 459** (0.7%↑)	**0. 918** (3.2%↑)	**0. 384** (1.8%↓)

Bold values are the results of our experimental method.

The symbols “↑” means the increase values compared to the previous method, "↓" means the decrease values compared to the previous method, and "✔" means the method used in the model.

#### 4.4.2 Effect of the WIoU loss

The loss function plays an important role in the deep learning training process. To further validate the performance of the proposed WioU loss, we conduct experiments comparing IoU, GioU, and CioU (Zheng et al., 2020). We present the comparison results in **Table 5**. We can find that GioU, CioU, and WioU make further detection improvements than the original IoU loss for most cases—for instance, the WheatFormer with WioU loss obtains 0.452 *mAP* , which is 2.9% higher than the IoU-based model, 1% higher than the GioU-based model, and 2.4% higher than the CioU-based model. Therefore, we can conclude that the WheatFormer can obtain better detection performance when trained with WioU loss.

**Table 5:** Results of WheatFormer with different IoU loss functions.

Method	IoU	GioU	CioU	WioU	*mAP*	*AP*_50_	*AP*_75_
WheatFormer	**✔**				0.423	0.894	0.322
		**✔**			0.442	0.896	0.374
			**✔**		0.428	0.900	0.326
				**✔**	**0.459**	**0.918**	**0.384**

Bold values are the results of our experimental method.

The symbols "✔" means the method used in the model.

#### 4.4.3 Performance of the variant models

As mentioned, we constructed three different variants of WheatFormer, and the detection results are shown in **Table 6**. WheatFormer-S obtains 42.4 MB parameters, similar to the Swin Transformer-based FCOS (43.8 MB), while WheatFormer achieves 0.438 at *mAP* (1.4% lower than SSD) and 0.908 at *AP*_50_ (2.2% higher than Swin Transformer-based FCOS). WheatFormer-B obtains 60.1 MB parameters, the same as Mask R-CNN. Nevertheless, our model achieves 0.459 at *mAP* (9.3% higher than Mask R-CNN) and 0.918 at *AP*_50_ (10.6% higher than Mask R-CNN), which significantly surpasses the detection ability of Mask R-CNN. The large version obtains parameters of 100.6 MB, showing a better performance than the previous versions.

**Table 6:** Comparison of variant models.

Method	*mAP*	*AP*_50_	*AP*_75_	*AP*_*m*_	*AP*_*l*_	#Params (M)
WheatFormer-S	0.438	0.908	0.366	0.402	0.516	42.4
WheatFormer-B	0.459	0.918	0.384	0.415	0.533	60.1
WheatFormer-L	0.466	0.927	0.400	0.422	0.524	100.6

### 4.5 Limitations and future work

In this work, we conduct extensive experiments to evaluate the effectiveness of the proposed methods. The experimental results prove that the proposed methods can greatly improve the detection performance of wheat spike detection. Although WheatFormer has shown to be effective in wheat spike detection tasks, there are still some limitations. It is worth noting that the experiment is only perfomed on the WSD-2022 dataset with a limited number of images. Moreover, our method attempts to improve the detection ability of the spike detector, while the parameters of our base model are relatively large. In future research, we will focus on solving the above-mentioned problems. Firstly, we will collect more wheat spike images containing more regions and more growth cycles to validate our methods. Secondly, we will continue to design more lightweight models to improve the capabilities for practical applications.

## 5 Conclusions

In this paper, we explore a Transformer-based network for wheat spike detection within a newly constructed dataset. We are the first to introduce the Transformer for wheat spike detection. To extract global and long-range semantic information, we design the MW-Swin Transformer as the backbone, and we propose the WioU loss function to improve positioning accuracy. Finally, we created a wheat spike dataset named WSD-2022 to verify the effectiveness of our model. The extensive experiments show that the method proposed in this study can obtain an encouraging detection performance compared with those state-of-the-art algorithms. We hope that this research will provide novel insights into the development of more advanced detection methods in the agricultural field.

## Data availability statement

The original contributions presented in the study are included in the article/supplementary materials. Further inquiries can be directed to the corresponding authors.

## Author contributions

QZ: conceptualization, methodology, software, investigation, formal analysis, and writing—original draft. ZH: conceptualization, methodology, software, investigation, formal analysis, and writing—original draft. SZ: visualization and investigation. LJ, LW and RW: conceptualization, funding acquisition, resources, supervision, and writing—review and editing. All authors contributed to the article and approved the submitted version.

## Funding

This work was supported by the National Key R&D Program of China (2019YFE0125700) and the Natural Science Foundation of Anhui Province (2208085MC57).

## Conflict of interest

The authors declare that the research was conducted in the absence of any commercial or financial relationships that could be construed as a potential conflict of interest.

## Publisher’s note

All claims expressed in this article are solely those of the authors and do not necessarily represent those of their affiliated organizations, or those of the publisher, the editors and the reviewers. Any product that may be evaluated in this article, or claim that may be made by its manufacturer, is not guaranteed or endorsed by the publisher.
